# Human Papillomavirus: Current and Future RNAi Therapeutic Strategies for Cervical Cancer

**DOI:** 10.3390/jcm4051126

**Published:** 2015-05-21

**Authors:** Hun Soon Jung, Nirmal Rajasekaran, Woong Ju, Young Kee Shin

**Affiliations:** 1 Research Institute of Pharmaceutical Science, Department of Pharmacy, College of Pharmacy, Seoul National University, Seoul 151-742, Korea; E-Mails: hunsoonjung@abionbio.com (H.S.J.); nirmalpharma@gmail.com (N.R.); 2 ABION Inc., R&D Center, 9th Fl., HanWha Biz Metro Bldg., 242 Digital-ro, Guro-gu, Seoul 152-733, Korea; 3 Department of Obstetrics and Gynecology, School of Medicine, Ewha Womans University, Seoul 158-710, Korea; E-Mail: goodmorning@ewha.ac.kr

**Keywords:** HPV E6 and E7 oncogenes, siRNA pool, cervical cancer

## Abstract

Human papillomaviruses (HPVs) are small DNA viruses; some oncogenic ones can cause different types of cancer, in particular cervical cancer. HPV-associated carcinogenesis provides a classical model system for RNA interference (RNAi) based cancer therapies, because the viral oncogenes E6 and E7 that cause cervical cancer are expressed only in cancerous cells. Previous studies on the development of therapeutic RNAi facilitated the advancement of therapeutic siRNAs and demonstrated its versatility by siRNA-mediated depletion of single or multiple cellular/viral targets. Sequence-specific gene silencing using RNAi shows promise as a novel therapeutic approach for the treatment of a variety of diseases that currently lack effective treatments. However, siRNA-based targeting requires further validation of its efficacy *in vitro* and *in vivo*, for its potential off-target effects, and of the design of conventional therapies to be used in combination with siRNAs and their drug delivery vehicles. In this review we discuss what is currently known about HPV-associated carcinogenesis and the potential for combining siRNA with other treatment strategies for the development of future therapies. Finally, we present our assessment of the most promising path to the development of RNAi therapeutic strategies for clinical settings.

## 1. Introduction

Human papillomaviruses (HPVs) are small DNA viruses with a genome size ~8 kb long. They infect cutaneous or mucosal epithelial cells, genital tissues, and the upper respiratory tract. To date, over 200 genetically distinct subtypes of HPV have been identified, and approximately 90 genotypes have been fully characterized. Among these types, the high-risk HPVs (HR-HPVs), including HPV-16, 18, 31, 33, 35, 39, 45, 51, 52, 56, 58, 59, 68, 73, and 82, are associated with more than 90% of cervical cancers, and to a lesser extent with other anogenital cancers and head and neck cancers [1,2,3,4]. Moreover, HPV-16 is associated with a small number of head and neck neoplasias, particularly tonsillar and oro-pharyngeal cancers [5]. Low-risk HPVs (LR-HPVs), including HPV-6, 11, 40, 42, 43, 44, 54, 61, 70, 72, and 81, have been linked to benign epithelial lesions [6]. In contrast to HR-HPV, infections with LR-HPV types 6 and 11 are associated with genital warts, essentially all laryngeal papillomas, and recurrent respiratory papillomatosis [7]. Over 50% of HPV-positive cervical cancers are associated with HPV-16, followed by HPV-18 (12%), HPV-45 (8%), and HPV-31 (5%) [8,9]. The HPV genome is composed of an early region (E) that encodes open reading frames involved in the regulation of viral replication and the viral life cycle, and a late region (L) that encodes two ORFs (L1 and L2) that form the viral capsid. During the course of HPV-mediated cancer development, the viral DNAs are frequently integrated into host cell chromosomes, and the proteins encoded by the viral genes play a critical role in carcinogenesis.

## 2. HPV and Cervical Cancer

**Figure 1:**
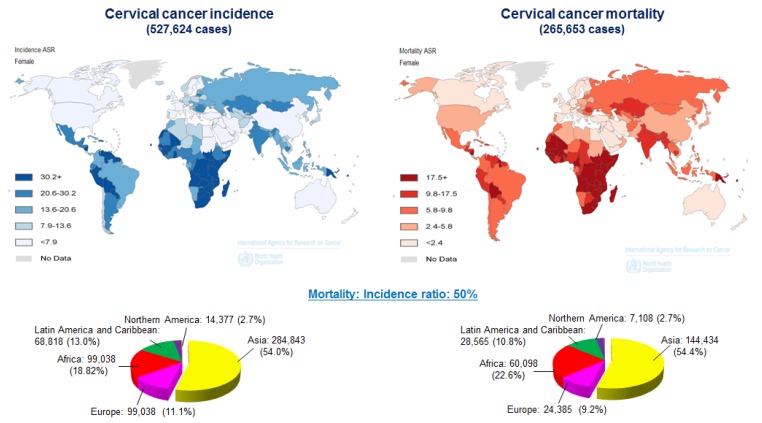
Worldwide cervical cancer incidence and mortality in 2012. Geographical distribution of cervical cancer incidence (**left)** and mortality (**right**) worldwide. Approximately 85% of cases occur in developing countries. Mortality: incidence rate ratios for cervical cancer were obtained from GLOBCAN (http://globocan.iarc.fr/).

Cervical cancer is one of the most common types of gynecological malignancies worldwide. According to the World Health Organization, the global prevalence of HPV infections in 2012 was approximately 630 million cases. Of these, 190 million were clinical infections leading to 528,000 new diagnoses of cervical carcinoma and ~266,000 deaths (Figure 1) (http://globocan.iarc.fr) [10]. Advances in research continue to improve the precautionary methods available in developed countries. Clinical and molecular epidemiological studies have clearly demonstrated that the major cause of cervical cancer is infection with HR-HPVs, such as types 16 and 18. The proteins encoded by HPV *E6* and *E7* are the primary oncogenes that play critical roles in HPV-positive cervical carcinomas. Inactivation of tumor suppressor protein p53 (TP53) and retinoblastoma (RB) is one of the main mechanism by which E6 and E7 induce carcinogenesis. Moreover, expression of the HR-HPV E6/E7 genes directly contributes to malignant progression by subverting genomic stability, which is also necessary for the induction of premalignant alterations [11].

### The Role of E6 and E7 in Carcinogenesis

The HPV E6 protein is a small basic polypeptide of approximately 150 amino acids that contains two zinc-finger motifs [12,13], each consisting of a CXXC-X_29_-CXXC sequence and the PDZ-binding epitope at *C*-terminal [14,15]. Overexpression of the E2 protein in HPV18-positive cervical carcinoma cells can negatively affect E6/E7 expression and inhibit cell proliferation [16,17]. In some types, further regulation is achieved through splice donor sites that give rise to truncated forms of E6, denoted as E6*I~IV, dependent upon the position of the downstream splice acceptors [18,19]. The most abundant splice RNA, E6*I, actually function as an E7 mRNA for efficient E7 translation [20,21,22]. The HR-HPV E6 protein and the truncated E6*I peptide destabilize several host proteins involved in cell growth and differentiation [19,23]. Oncogenic HPV infection also deregulates the expression of oncogenic and tumor suppressive miRNAs via E6-TP53 and E7-pRb pathways [24,25].

After viral integration, E6 is expressed, and it facilitates several cellular changes that prolong the cellular lifespan by blocking apoptosis and increasing telomerase activity. The transcriptional activator role of E6 may be coupled to its ability to immortalize and transform cells [26]. E6 binds to cellular proteins, particularly to the HECT domain of ubiquitin ligase E3A (UBE3A), alternatively known as E6-associated protein (E6AP), and to E6BP (reticulocalbin 2, an EF-hand calcium-binding domain). E6BP is a 320 amino acid protein with a high affinity for calcium. E6 can bind to E6BP in the absence of TP53. UBE3A interacts with HPV E6 at a conserved LXXLL motif and forms ternary complexes with TP53, which are then degraded through ubiquitin-dependent mechanisms [27,28,29]. Moreover, E6 can block the translocation of TP53 into the nucleus [30] and thereby inhibit the gene expression regulatory functions of TP53. The consequence of TP53 degradation and blocking of TP53 transport into the nucleus disrupts TP53-mediated cell cycle control, allowing continued cell division despite DNA damage. HR-HPV E6 can also impair apoptosis by accelerated degradation of Bak, c-Myc, FADD, and procaspase-8. Furthermore, E6 binds to E6TP1, hADA3, tuberin, CBP/p300, and Gps2, interfering with the function of these proteins to negatively regulate cell proliferation. HPV also suppresses the innate immune system through binding of E6 to IRF-3, as well as E6-mediated downregulation of TLR9 expression [31]. Also oncogenic HPV E6 is capable of regulating the expression of many cellular miRNAs like miR-34a [32].

E7 ORF encodes an acidic phosphoprotein with zinc-binding motifs consisting of CXXC-X_29_-CXXC [33], which are essential for proper protein folding and stability. The E7 protein is primarily localized to the nucleus and has been shown to induce cellular proliferation, immortalization, and transformation [34]. HR-HPV E7 confers transforming activities and can immortalize human keratinocytes via interactions with factors involved in the regulation of cell growth [35]. Most E7 proteins contain a strictly conserved LXCXE-binding motif that associates with members of the RB family of tumor suppressors, resulting in ubiquitin-mediated targeted degradation of the associated RB family members [36]. Binding of E7 to hyper-phosphorylated RB (pRB) results in the release of the E2F transcription factor, activating gene transcription [37]. In addition, HR-HPV E7 mediates the degradation of pRB [38]. Both HR and LR-HPVs possess the ability for E7 to bind to pRB, although the interaction between LR-HPVs E7 and pRB is much weaker [39]. Furthermore, HR-HPV E7 not only interacts with cyclins A and E, but also increases the levels of these proteins. In addition, E7 expression results in inactivation of the cyclin-dependent kinase (CDK) inhibitors CDKN1A (p21^CIP1^) and CDKN1B (p27^KIP1^) [37]. HPV-16 E7 protein can modulate the cytoplasmic localization of CDKN1B (p27^KIP1^) and may, in turn, regulate tumor metastasis/aggressiveness through the PI3K/AKT pathway [40]. E7 proteins act by binding with histone deacetylases, which are normally recruited to repress transcription via promoters containing E2F-binding sites. 

In addition, a synergistic effect between Ras and E6/E7 genes in cellular transformation has been reported [41,42,43]. E6/E7 expression can activate PI3K and MAPKs, particularly ERK1 and ERK2 [44]. It appears that several transcription factors contribute to HPV-mediated carcinogenesis, including the TATA box-binding protein (TBP), a component of the NURD histone deacetylase complex, Mi2β [45,46], the acetyl transferases p300/CBP and p300/CBP-associated factors (P/CAF) [47], and E2F [48]. Thus, the E6 and E7 oncoproteins are thought to immortalize cells, primarily through interference with the TP53 and pRB tumor suppressor proteins. In addition, cervical cancer displays notably increased or decreased expression of a large number of cellular oncogenic or tumor suppressive miRNAs. The elevated expression of miR-16, miR-25, miR-92a, and miR-378 and the decreased expression of miR-22, miR-27a, miR-29a, and miR-100 were attributed to viral oncoprotein E6 or E7 [24,25]. These observations are not surprising because both E6 and E7 modulate numbers of major transcription factors such as MYC, TP53, and E2F, which are upstream molecules for a large number of these miRNA genes.

## 3. TP53 Mutations in Human Cancer

TP53 is the most commonly mutated gene in human cancer [49]. Genetic alterations of *TP53* in human tumors include allelic losses, missense, frame-shift mutations and intragenic deletions, and epigenetic changes are also observed. Our analysis of 17,584 tumor samples with a variety of human cancer types in The Cancer Genome Atlas (TCGA) database showed that the *TP53* gene was frequently mutated in ovarian (90.66%), uterine carcinosarcoma (89.5%), esophageal (71.9%), head and neck (70.18%), lung (60%), colorectal (54.14%), and other cancers. In the case of cervical cancer, mutations in the TP53 gene are rarely reported, with an occurrence of only 5.1%. Tumor suppression by TP53 is primarily regulated through Mdm2-mediated ubiquitination of TP53. However, in HPV-positive cervical cancer cells, the degradation of TP53 is completely converted from Mdm2 to E6-mediated ubiquitination [50]. Thus, the level of TP53 protein in cervical carcinomas remains remarkably low, despite TP53 signaling activation, including oncogenic addiction and DNA damage by reactive oxygen species (ROS). Therefore, most HPV-associated cervical carcinomas, unlike many other cancers, usually carry the wild-type (WT) *TP53* gene [51,52]. Here, we also focus on the role of TP53 and the implications of TP53-based anticancer therapies for cervical cancer.

## 4. Therapeutics against HPVs

In 2006, the U.S. Food and Drug Administration approved Gardasil, the first quadrivalent cancer vaccine, for use in women 9–26 years of age for the prevention of cervical cancer, precancerous genital lesions, and genital warts caused by HPV6, HPV11, HPV16, and HPV18 [53,54]. In 2009, FDA approved Cervarix, another vaccine to prevent cervical cancer and precancerous lesions caused by human papillomavirus (HPV) types 16 and 18. The vaccine is approved for use in girls and women ages 10 years through 25 years. However, current vaccine efficacy is not evident for the therapy of cervical cancer patients.

For cervical cancer therapy, E6 or the E6/UBE3A complex deserves special attention as a specific target. In Table 1, we discussed several strategies that target E6 or the E6/E6-AP complex that have been developed, including various therapies that employ cytotoxic drugs, a zinc-ejecting inhibitor of the viral E6 oncoprotein, an E6-AP mimetic epitope peptide (mimotope), an anti-E6 ribozyme, peptide aptamers that target the viral E6 oncoprotein, siRNAs that target the viral E6 oncogene, and combinations of all these therapies [55,56,57,58,59,60,61,62,63]. Based on recent reports a possible new strategy is to induce viral E6 and E7 instability by using HSP90 and GRP78 inhibitors for the treatment of cervical cancer [64]. An E7 antagonist peptide has been studied to determine its therapeutic efficacy. The E7 antagonist showed antitumor effects through pRB reactivation both *in vitro* and *in vivo* [65]*.* GS-9191, a nucleotide analog prodrug, showed an antiproliferative effect *in vitro*, and its topical application reduced the size of papillomas in the cottontail rabbit papillomavirus model [66]. Chitosan hydrogel containing granulocyte-macrophage colony-stimulating factor (GM-CSF) in combination with anticancer drugs, cyclophosphamide in particular, results in antitumor effects through CD8+ T cell immunity [67]. Heparin-like glycosaminoglycans have been shown to inhibit tumor growth by the downregulation of HPV18 long control region activity in transgenic mice [68]. Finally, 5-aza-2′-deoxycytidine, a demethylating agent, and 5,6-dimethyl xanthenone-4-acetic acid, a vascular disrupting agent, have each been combined with therapeutic HPV DNA vaccines [69,70], showing significant antitumor therapeutic effects *in vivo*. 

**Table 1:** HPV-targeting therapeutics.

Reagent		References	Note
HPV E7 antagonist	Peptide	[65]	
E6-binding aptamer	Peptide	[58]	
E6-AP mimetic epitope	Helical peptides	[60]	
Organic disulfides containing dithiobisamine moiety	Organic compound	[55]	
Anti-E6 ribozyme	RNA molecule	[63]	
GS-9191	Nucleotide analog prodrug	[66]	
5-aza-2′-deoxycytidine (DAC)	Nucleotide analog	[69]	Combination with therapeutic HPV DNA vaccine
5,6-dimethylxanthenone-4-acetic acid (DMXAA)	Small molecule	[70]	
Cisplatin	Platinum compound	[61]	Alone or combination treatment
4,4′-dithiodimorpholine	Zinc-ejecting inhibitor	[55]	
Glycosaminoglycans (GAGs)	Heparin-like	[68]	
Chitosan hydrogel	Natural biopolymer	[67]	Combination treatment
Methyl jasmonate	Plant hormone	[72]	Plant-originated products
Epigallocatechingallate (EGCG)	Plant-derived natural compound	[78]	
Nordihydroguaiaretic acid (NDGA)	Plant lignan derivative	[80]	
Silymarin	Plant flavonoid	[76]	
Jaceosidin (4′,5,7-trihydroxy-3′,6-dimethoxyflavone)	Plant-derived natural compound	[79]	
Withaferin A	Plant-derived natural compound	[77]	
Praneem tablet	Plant extract	[71]	
Curcumin	Plant extract	[75]	
Phytoglycoprotein	Plant-originated glycoprotein	[74]	
Soyasaponins	Plant-derived natural compound	[73]	
Carrageenan	Compound from red algae	[81]	
17-*N*-allylyamino-17-demethoxygeldanamycin (17-AAG)	A derivative of the antibiotic geldanamycin	[64]	Combination treatment with GRP78 inhibitor

Additionally, several plant-derived compounds have been investigated to determine their therapeutic potential in cervical cancer (Table 1). In clinical trials, intravaginally administered Praneem, a polyherbal formulation, was shown to eliminate HPV16 infection in early cervical intraepithelial lesions [71]. Methyl jasmonate, soyasaponin, phytoglycoprotein, curcumin, silymarin, withaferin A, and epigallocatechingallate (EGCG) showed therapeutic effects via repression of viral oncogenes, upregulation of tumor suppressor genes, or induction of apoptosis *in vitro* [72,73,74,75,76,77,78]. Withaferin A treatment reduced tumor volume by approximately 70% in a xenograft model. Interestingly, a few of these natural compounds specifically target HPV. For example, jaceosidin inhibits the functions of the E6 and E7 oncoproteins in HPV16-positive cervical cancer cells [79], and nordihydroguaiaretic acid also inhibits HPV16 gene expression [80]. Carrageenan, a compound produced by red algae, inhibits HPV infection through direct binding to the viral capsid, and through a heparan sulfate proteoglycan-independent inhibitory effect [81].

### 4.1. RNAi-based Therapeutics against HPVs

Recently, novel antiviral RNAi therapies have been developed and tested in clinical trials with short interfering RNAs (siRNAs) [82]. siRNAs have been demonstrated to be capable of selective silencing of endogenous genes in mammalian cells [83,84], and of selectively silencing viral genes in virus-induced diseases [85,86,87]. Remarkably, it has been reported that RNAi targeting of E7 or E6/E7 promotes the accumulation of TP53 and/or pRB, eventually leading to the induction of apoptosis and/or senescence in HPV16-positive cervical cancer cell lines [59,62,88,89], as well as in HPV18-positive human cervical cancer cells [90,91]. It has been reported earlier that, silencing of both E6/E7 produces greater anticancer activity than E6 alone [92,93,94]. Several studies of RNAi targeting of the HPV E6 or E7 oncogenes are summarized in Table 2.

**Table 2:** List of RNAi studies targeting HPV E6 or E7.

	References	Cell line		Target Transcripts	Note
Cun *et al.*, 2013	[149]	OCM1, OM431, VUP and SP6.5	Synthesized siRNA	E6 & E7 mRNA	
Li *et al*., 2013	[150]	SiHa	Plasmid-based E6-specific siRNA	E6 mRNA	Dual coexpressed-E6-specific siRNA and wild type TP53
Zhou *et al*., 2012	[93]	SiHa	Synthesized siRNA	E6/E7 mRNA	BALB/C nude mice, intratumoral injections every other dayduring a 12-day period
Jung *et al*., 2012	[94]	HeLa, CaSki, SiHa	Synthesized siRNA	E6/E7 mRNA	BALB/C nude mice, intravenous injections in combination with Cisplatin
Chang *et al*., 2010	[95]	CaSki, HeLa	siRNA Plasmid	E6 only or E6/E7 mRNA	BALB/C nude mice, intratumoral injections twice a week for two weeks
Hong *et al*., 2009	[96]	SiHa	Synthesized siRNA	E6/E7 mRNA	
Jonson *et al*., 2008	[97]	CaSki	Synthesized siRNA	E6/E7 mRNA	nu/nu mice, intratumoral injections every three days for 35 days
Sima *et al*., 2008	[88]	SiHa, CaSki	shRNA	E6/E7 mRNA	
Lea *et al*., 2007	[98]	HeLa	Synthesized siRNA	E6 only or E6/E7 mRNA	
Courtete *et al*., 2007	[99]	CaSki, SiHa, HeLa	Synthesized siRNA	E6 mRNA	
Fujii *et al*., 2006	[100]	SKG-IIIa, SKG-II, HeLa	Synthesized siRNA	E6 & E7 mRNA	Nude mice, Intratumoral infections for 10 days
Tang *et al*., 2006	[20]	CaSki and SiHa, HeLa	Synthesized siRNA	E6 or E6*I mRNA and E7 mRNA	
Yamato *et al*., 2008	[89]	CaSki and SiHa,	Synthesized siRNA	E6 mRNA	
Koivusalo *et al*.,2005	[101]	HeLa	Synthesized siRNA	E6 mRNA	siRNA combination with different drugs
Putral *et al*., 2005	[102]	SiHa, CaSki	Synthesized siRNA	E6* mRNA and full length E6 mRNA	
Yoshinouchi *et al*., 2003	[62]	SiHa	Synthesized siRNA	E6* mRNA and full length E6 mRNA	
Butz *et al*., 2003	[58]	HeLa	Vector and Synthesized siRNA	full length E6 mRNA	
Hall and Alexander, 2003	[91]	HeLa	Synthesized siRNA	E7 mRNA	
Jiang and Milner, 2002	[59]	CaSki, SiHa	Synthesized siRNA	E6 and E7 mRNA	

Zhou *et al*. [93] recently reported that two siRNAs targeting the E6/E7 promoter and E7 transcripts produced E6 and E7 mRNA knockdown, increased TP53 protein levels, decreased CDKN2A (p16^INK4A^) protein levels, and exhibited SiHa cell growth inhibition via apoptosis. In xenografted mice, siRNA transfection of cells *in vitro* or intratumoral siRNA injection also inhibited tumor growth and induced apoptosis. Another study reveals that HPV16 E6/E7 silencing by this promoter-targeting siRNA was related to histone modification associated with histone H3-Lys9 methylation [96]. Chang *et al*. [95] designed nine siRNAs that specifically targeted E6 or E6/E7 mRNA of HPV16 or HPV18. Intratumoral administration of potent siRNA resulted in the inhibition of tumor growth and induction of apoptosis *in vivo*, suggesting that siRNA treatment shows potential as an adjuvant therapy for cervical cancer. The therapeutic mechanism of siRNA-mediated silencing of both E6 and E7 mainly depends on the reactivation of TP53 and pRB, inducing apoptosis and strong cellular senescence. In our recent study, anti-angiogenesis and inhibition of lung metastasis were determined to constitute additional mechanisms operating in combination therapy with E6/E7-specific siRNA and cisplatin [94]. Hall *et al*. [91] examined an siRNA sequence that targets the type I transcript of E6/E7 mRNA and found reduced levels of full-length E6 and E7 mRNA in cells. They confirmed that this siRNA sequence transfected cells show reduced E6 mRNA levels, leading to increase of the expression level of TP53 protein. They also demonstrated that transfection with E6/E7-targeting siRNA inhibited DNA replication and induced cellular senescence in HPV 18-positive human cervical cancer cells. Hall *et al*. [91] also suggested that siRNA inhibition of E6 and E7 demonstrates potential as a therapeutic intervention for the treatment of cervical cancer. In support of this proposition, Jonson *et al*. [97] confirmed the therapeutic effect of direct intratumoral injection of HPV16 E6/E7 siRNA using the CaSki xenograft model. 

### 4.2. Anticancer Therapeutic Strategies Targeting Activation of the TP53 Pathway

In cervical cancer, the TP53 pathway is often disrupted. Therefore, functional restoration of WT-TP53 may induce the regression of cervical carcinomas, which can be achieved by abrogating the expression of the E6 or E6/E7 oncogenes, or through cisplatin (*cis*-diaminedichloroplatinum II; CDDP) or radiation treatment. It has been demonstrated that cisplatin therapy allows TP53 to escape from E6-mediated degradation, thereby facilitating TP53 accumulation in the nucleoli of HeLa cells [61]. Concurrent treatment with cisplatin and radiotherapy also restores TP53 function and enhances the radiosensitivity of HPV16-positive SiHa cells [103]. Additional reports have arrived at the same remarkable conclusion [104,105,106]. The restoration of TP53 function in established tumors (including lymphomas, sarcomas, and hepatocellular carcinomas) causes regression of tumors *in vivo* and could represent an effective new approach to treating cancer. Evans *et al.* [104] used the Eμ-myc mouse model to validate the effects of modulation of the TP53 pathway on various aspects of tumor biology. They developed a TP53 knock-in model, in which the WT-*TP53* gene is replaced by a chimeric TP53 protein containing an estrogen receptor (ER) domain linked to the WT-TP53 sequence. TP53 restoration markedly increased tumor cell death and significantly prolonged survival of the mice. Jacks *et al.* utilized a mouse model, in which TP53 was restored by removal of the STOP cassette following activation of a tamoxifen-inducible Cre recombinase. Interestingly, in lymphomas, TP53 restoration induces apoptotic cell death; whereas, in sarcomas, it induces cell-cycle arrest with signs of cellular senescence [105]. 

Lowe *et al.* developed a mouse model of hepatocellular carcinoma [106]. In this study, purified embryonic liver cells were transformed by introduction of an activated H-ras oncogene, and TP53 expression was downregulated using a doxycycline-repressible short hairpin RNA directed against TP53. TP53 induction in this hepatocarcinoma model led to growth arrest with senescence rather than apoptotic cell death. In addition, the efficiency of TP53-mediated tumor clearance is stage-specific and dependent on the overall heterogeneity and aggressiveness of tumor cell populations [107,108]. Silencing or reactivation of TP53 may also regulate the expression level of host microRNAs regulations in cervical cancer [32,109]. Numerous other recent approaches investigating TP53 protein dynamics have provided new insights into the topology of the signaling network. The majority of studies revealed clear differences in gene expression when TP53 is pulsing and when TP53 expression is sustained at high levels for several hours. In response to ultraviolet light, TP53 shows a single graded pulse; whereas, in response to double-strand breaks caused by γ irradiation, TP53 exhibits a series of repeated pulses [109,110]. TP53 was also activated following the administration of various anticancer chemotherapeutic agents [111]. However, the ability of a TP53 pulse to arrest the cell cycle and activate its target genes, such as CDKN1A, depends on specific posttranslational modifications, which occur only in response to severe extrinsic cell damage [112]. Depending upon its expression level, TP53 can promote either DNA damage repair or survival of damaged cells. In recent years, another treatment method has emerged, in which the TP53 pulses are sustained by Nutlin-3, an MDM2 inhibitor that can be employed alone or in combination with chemo/radiotherapy; this appears to be a valid approach for the treatment of tumors [113,114]. Sustained induction of TP53 triggers multiple cellular programs ranging from transient responses, such as DNA repair and cell cycle arrest, to terminal fates, such as apoptotic cell death and senescence. As an alternative strategy, stabilization of TP53 by Nutlin-3a significantly enhanced reovirus-induced apoptosis [115]. Overall, combinations of conventional therapies can induce frequent TP53 pulses and sustain their expression level, leading to irreparable damage and apoptosis of cancer cells.

Our unpublished live cell imaging results on reporter activity also revealed that combination of a HPV E6/E7 siRNA pool with a chemotherapeutic agent sustained TP53 dynamics over a long period, and led to immediate cell death. Analyzing the TP53 dynamics can be an important part of a signal in cancer, directly influencing different functional cellular outcomes, such as growth, survival, and death. Overall, stabilization and maximal activation of the TP53 signaling network can facilitate determination of the optimal therapeutic strategy for cervical cancer.

## 5. RNAi-based Combination Therapeutics against HPV

Several RNAi studies have been performed in human tumor cell lines using synthetic siRNAs or short hairpin RNA (shRNA) libraries to identify modulators of drug sensitivity [116,117,118,119,120,121,122]. These studies address whether combination therapy with siRNA may significantly enhance the sensitivity of cancer cells and may help to prevent chemo/radio resistance resulting from low-dose chemo/radio therapy. From a clinical perspective, if the combined effect is equal to the sum of the effects of the individual modalities, then the effect of the multiple modalities is additive. If the combined effect is greater than the predicted effect of the multiple individual modalities, however, then the interaction is synergistic. The multiple modalities may also interact in an antagonistic fashion. Statistical analysis using the Chou-Talalay method must be applied in order to determine the synergistic, additive, or antagonistic effects of RNAi-based combination therapies. 

In our study, we validated the role of 2-in-1 antiviral siRNAs targeting the HPV E6 and E7 oncogenes as a potential sensitizer to CDDP [94] and radiation therapy (unpublished) for the treatment of cervical carcinoma. Thus, the combination of siRNAs targeting E6 or E6/E7 may have synergistic effects on the restoration of TP53 and/or pRB function, and may be a more effective therapeutic modality for the treatment of cervical cancer. Putral *et al.* [102] investigated CDDP co-therapy, and found that shRNAs to E6 increased CDDP sensitivity in HeLa cells. Tang *et al.* [92] also reveals the silencing of HPV16 E6 and E7 expression by the shRNA in cervical cancer cells; at late passages the cells develop a resistant program in the presence of E7 siRNAs. Another recent study revealed that, intratumoral injection of both exonic (E6/E7-Exon) and intronic (E6/E7-Intron) siRNAs co-administration with intravenous injection of paclitaxel treatment restored the tumor-suppressive effect more effectively than co-administration of paclitaxel treatment with (E6/E7-Exon) siRNA. Also, both E6/E7 siRNA co-administrations with paclitaxel treatment resulted in similar tumor-suppressive effect compared with paclitaxel alone, and showed some extended survival [123]. Further studies are required to overcome all the key points mentioned above and to develop clinically applicable combination therapies based on RNAi. Most importantly, to establish proof-of-concept for RNAi-based combination therapeutics, the mechanism underlying the synergy between the treatments should be elucidated. The results of *in vivo* experiments have demonstrated that combination therapy is significantly superior to either modality alone. Moreover, assessments to verify the absence of off-target effects and minimal induction of interferon are a prerequisite for the clinical application of RNAi-based combination therapeutics. 

## 6. Unmet Medical Needs of Cervical Cancer

In the 1990s, the Gynecologic Oncology Group (GOG) conducted a randomized trial for radiotherapy in combination with three concurrent chemotherapy regimens—CDDP alone (group 1); CDDP, fluorouracil, and hydroxyurea (group 2); and hydroxyurea alone (group 3)—in patients with locally advanced cervical cancer (stage IIB, III, or IVA without involvement of the para-aortic lymph nodes) [124]. The groups receiving CDDP (groups 1 and 2) showed a higher rate of progression-free survival (PFS) than the group receiving hydroxyurea alone (group 3). The overall survival (OS) rate was also significantly higher in groups 1 and 2 than in group 3. Moreover, the highest combined frequency of grade 3 (moderate) and grade 4 (severe) adverse effects was observed in group 2, which means that radiation in combination with CDDP produced fewer side-effects than with the other triple agent combination. As a result, CDDP has been proposed for use as a standard radiotherapy and chemotherapy drug for locally advanced cervical cancer based on that trial. 

Morris *et al.* [125] compared the effect of administering radiotherapy to a pelvic and para-aortic field with that of administering pelvic radiation in combination with chemotherapy (fluorouracil and CDDP) in women with advanced cervical cancer (stages IIB through IVA or stage IB or IIA with a tumor diameter of at least 5 cm or involvement of the pelvic lymph nodes). Kaplan-Meier analysis revealed that disease-free survival (DFS) and OS rates were significantly higher in patients treated with radiotherapy and chemotherapy than in those treated with radiotherapy alone. They found that the addition of chemotherapy to radiation treatment significantly improved survival among women with locally advanced cervical cancer. In the combined therapy group, the reported rate of local relapse was 19%, and the rate of distance relapse was 14%. Keys *et al.* [126] determined that weekly infusions of CDDP during radiotherapy improved PFS and OS among patients with bulky stage IB cervical cancer. They also found that chemoradiotherapy followed by radical hysterectomy significantly reduced the risk of disease recurrence and death in women with bulky stage IB cervical cancers. However, the chemoradiotherapy group exhibited higher frequencies of transient grade 3/4 adverse hematologic effects and adverse gastrointestinal effects. Furthermore, although residual tumors were not more frequently detected in the combined-therapy group than in the radiation alone group, residual masses in the combined-therapy group were reported as often as 48%. 

**Figure 2:**
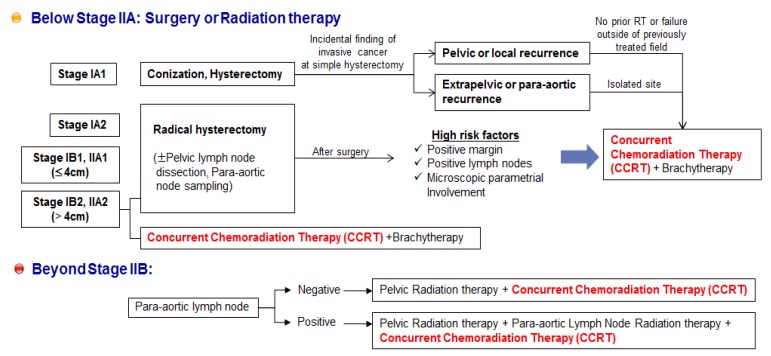
Practice guideline summary for cervical cancer. Concurrent chemoradiation therapy (CCRT) is recommended as the first-line therapy for patients with disease at stage IIB or greater, and as an adjuvant therapy following radical hysterectomy for patients with locally advanced cervical cancer.

**Figure 3:**
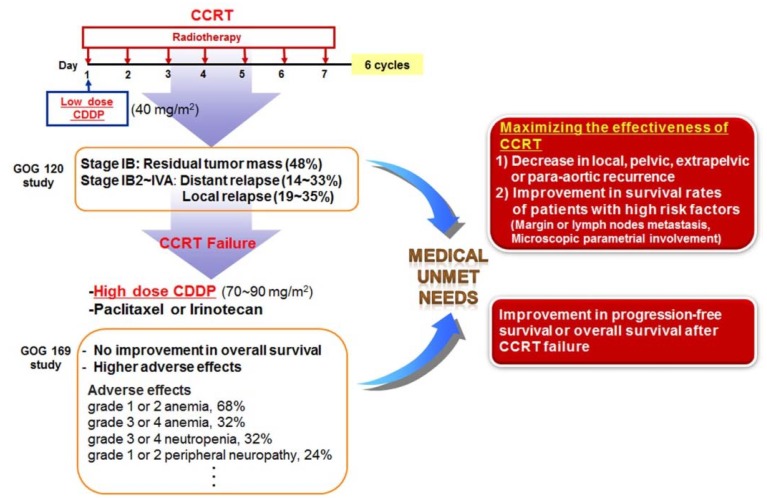
Unmet medical needs of cervical cancer. It is important to maximize the effectiveness of CCRT in order to decrease recurrence and improve the survival rate. Following failure of CCRT, the clinical endpoint is to improve progression-free survival, overall survival, and the reduction of severe adverse events.

Collectively, these randomized trials for cervical cancer all demonstrated that concomitant treatment with CDDP and radiotherapy produced more favorable outcomes than radiotherapy alone. Therefore, CDDP chemotherapy with concurrent radiation (CCRT) is recommended as the first-line therapy for patients with disease at stage IIB or greater, and as an adjuvant therapy following radical hysterectomy for patients with locally advanced cervical cancer (Figure 2) [125,126,127]. Although this practice guideline for cervical cancer has improved the survival rates for patients undergoing CCRT, CCRT failure still occurs, including residual cancers, adverse effects, local recurrences, or distance recurrences, as mentioned above [124,125,126]. In addition, when primary therapy with CCRT fails to treat cervical cancer, most patients may no longer be sensitive to radiation therapy alone or CDDP chemotherapy alone, and single-agent therapy cannot be considered as a secondary therapy following failure of CCRT. Thus, patients with recurrent or metastatic squamous cell carcinoma of the cervix receive high-dose CDDP plus paclitaxel combination therapy as a palliative chemotherapy [128,129] (Figure 3).

A phase III GOG study was conducted to determine whether CDDP plus paclitaxel improved response rate, PFS, or survival compared with CDDP alone in patients with stage IVB, recurrent, or persistent squamous cell carcinoma of the cervix [129]. The combination of CDDP and paclitaxel appears to be superior to CDDP alone with respect to the objective response rate, PFS, and sustained quality of life, but with increased adverse effects and no improvement in OS in patients with stage IVB, recurrent, or persistent squamous cell carcinoma of the cervix. Another GOG study reported that the combination of topotecan and CDDP produced superior response and OS compared to CDDP alone [130]. Therefore, unmet medical needs remain regarding two points for the treatment of cervical cancer (Figure 3). First, in cases of early lesions with high risk factors (margin or lymph node metastasis, or microscopic parametrial involvement) after surgery, and in advanced lesions beyond stage IIB, it is important to maximize the effectiveness of CDDP-based concurrent CCRT, in order to decrease recurrence and improve the survival rate.

Second, we must consider patients who experienced recurrence and metastasis following failure of CCRT. Clinical studies have been conducted for methods to improve PFS, OS, and to reduce severe adverse events. Although at present, CDDP is considered to be the most active cytotoxic agent available, various combinations of chemotherapy with paclitaxel, carboplatin, topotecan, irinotecan, gemcitabine, bevacizumab, or docetaxel have been employed in clinical studies of metastatic or recurrent cervical cancer to alleviate poor prognosis of recurrent and metastatic cervical cancer [128,129,130,131,132,133,134,135]. In summary, the evidence discussed here suggests that novel anticancer therapeutics, in combination with CCRT, show promise as therapies to control cervical cancer.

## 7. Potential Combination Therapies for Cervical Cancer

Radiation therapy constitutes the use of various forms of radiation to safely and effectively treat cancer. The ability of radiation therapy to provide both curative and palliative treatment has underscored its importance in cancer therapy. In the clinical setting, radiation can be delivered in a targeted manner to the area of interest, allowing for its specific delivery to tumor tissues. In the last few decades, technological advancements in the delivery of radiation and in treatment planning have resulted in improved patient outcomes, particularly in the reduction of normal tissue toxicity [136,137]. The generation of hydroxyl radicals is thought to be the initiating event that leads to the majority of the biological damage created by ionizing radiation. 

**Figure 4:**
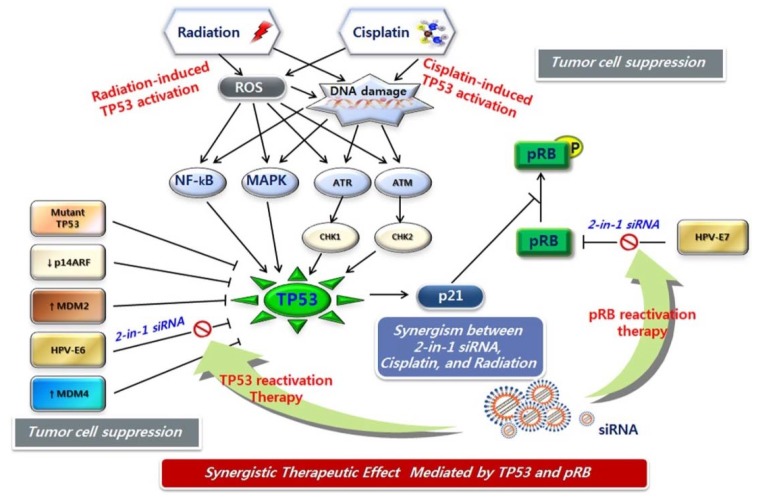
Dual activation of TP53 and pRB in combination therapy with CCRT and HPV E6/E7 siRNA. Cisplatin and radiation therapies induce TP53 activation by DNA damage signal transduction. HPV E6/E7 siRNA treatment results in TP53 and pRb reactivation. Combination therapy with CCRT and HPV E6/E7 siRNA may fully activate functional TP53 and pRb through different transduction mechanisms, leading to strong cellular response.

DNA damage can take the form of either single-strand breaks (SSBs) or double-strand breaks (DSBs). SSBs are more easily repaired by the cell, and hence are less likely to be mutagenic or lethal. DSBs are the most lethal DNA damage induced by radiotherapy. The ATM kinase initiates a signaling pathway that is primarily induced by DSBs and that acts by phosphorylating hundreds of proteins [138]. CHK2 is an important effector molecule targeted by ATM. The ATR kinase activates a pathway principally induced by UV damage that involves CHK1 kinase [139,140,141]. Among the targets of ATM and ATR is TP53, which plays a key role in controlling DNA-damage-induced G1/S and G2/M checkpoints. TP53 can activate CDKN1A, which facilitates cell cycle arrest and DNA repair, or induced apoptosis if repair is impossible. In addition, JNK and p38, members of the MAPK family, are highly activated in response to ionizing radiation. TP53 also interacts with several crucial components of the DNA repair machinery and is situated at a crossroads among radiation-induced DNA repair, apoptosis, and senescence (Figure 4). 

CDDP is the most active and well-known anticancer agent available for clinical use. The mechanism of action of platinum-based drugs was recently reviewed; however, the cellular processing of CDDP, including the regulation of drug uptake and efflux, and the signaling of DNA damage, cell-cycle arrest, DNA repair, and cell death, is still not fully understood [142]. CDDP-induced DNA damage signals through ATM/ATR activated cell cycle checkpoints (CHK1, CHK2) and leads to DNA repair, cell cycle arrest, and apoptosis, which are mediated by the TP53 pathway (Figure 4). CDDP also triggers the activation of the MAPK pathways in tumor cells. Following DNA damage, the ERK, JNK, and p38 MAPK cascades are activated; these subsequently activate downstream target genes, including TP53.

While both radiation therapy and chemotherapy have been used individually as treatment modalities in cancer patients for some time, the advantages of combined chemo-radiotherapy have only recently been observed clinically. The theoretical framework for the interaction between these two modalities was introduced in 1979 by Steel and Peckham [143]. Radiation therapy affects locoregional control of the tumor mass; whereas, chemotherapy acts on distant metastasis, with no interaction between the two modalities. In addition, chemotherapy co-operates with radiation therapy in the radiation field, leading to increased killing of cancer cells. As mentioned above, CDDP in combination with concurrent radiation is commonly recommended for patients with disease at stage IIB or greater, and for those with locally advanced cervical cancer. Recently, a breakthrough study demonstrated that TP53 was expressed in a wavelike or “pulsed” manner following exposure to ionizing radiation alone [144]. Sustained TP53 expression or an increased number of TP53 pulses increases the probability that TP53 will activate downstream targets involved in the induction of apoptosis and senescence. In addition, activation of the tumor suppressor pRB is part of the mode of action of HPV E6/E7 siRNA (Figure 4). Thus, full reactivation of TP53 is possible, and its dynamics are better sustained by combination therapy with CCRT and HPV E6/E7 siRNA. 

To fulfill the medical unmet needs in CCRT failure, further studies are required to optimize the alternative combined therapeutic strategies like HPV E6/E7 siRNA in combination with combined chemotherapy via intravenous delivery system for apoptosis and senescence assay validation, analysis of dynamic behavior of TP53, Chou-Talalay analysis, and other validations with better therapeutic outcome. In theory, combination with HPV E6/E7 siRNA therapy can benefit from synergistic effects and reduced toxicity because of the lowered dose of chemotherapy and/or radiotherapy required.

## 8. siRNA Pooling Technology, a Promising RNAi Therapy

Long double-stranded RNA (dsRNA) initiates RNAi, which can generate as transcripts from an invading virus, a transposon, or other inappropriately transcribed endogenous sequence. In mammalian cells, Dicer digests dsRNA into a pool of duplex siRNAs that bind with the RNA-induced silencing complex (RISC) to induce specific gene silencing. Naturally, the siRNAs generated by Dicer represent a pool of siRNAs that function together to silence the expression of the targeted gene. Thus, to replicate the process, pools of synthetic siRNAs are utilized to silence individual genes in a specific manner. A reduction in the silencing efficiency of siRNA pools was attributed to the saturation of RISC with non-functional siRNAs that combine with the more functional duplexes for access to the RNA silencing machinery. However, highly potent siRNAs, which are rationally designed, screened, validated for efficacy, and chemically modified for stability, can mimic the pools generated in nature. Because each siRNA has a strong therapeutic effect, this siRNA mixture (pooling technology) reduces the effective concentration of each siRNA and reduces the off-target effects. Therefore, siRNA pools also show potential for therapeutic benefit in HPV E6/E7 targeting-siRNA drug development. 

**Figure 5:**
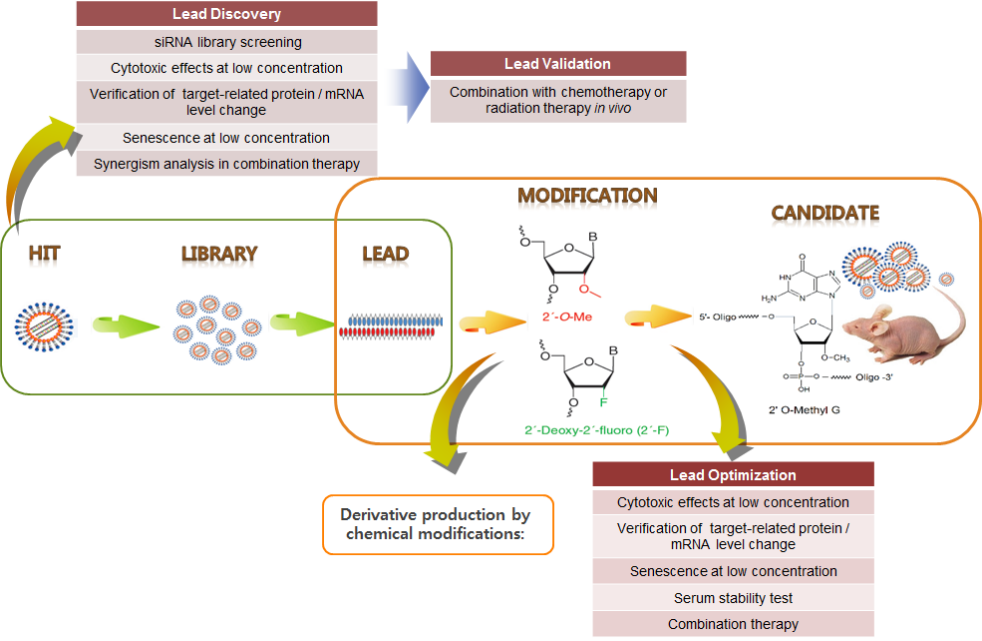
Example of HPV E6/E7 siRNA drug development pipeline. Following the RNAi therapeutics pipeline, highly potent siRNA leads are chemically modified to increase their stability and specificity. Following optimization, siRNA candidates can be selected.

Yamato *et al.* [145] screened an HPV16 siRNA library and identified several potent E6/E7 siRNAs. Subsequently, these potent siRNAs were modified as RNA–DNA chimeric siRNAs to enhance their specificity [146]. Previously, we identified and validated HPV E6/E7 siRNA hits and then established proof-of-concept of combination therapy with CDDP [94]. Following the RNAi therapeutics pipeline, HPV16- and 18-type siRNA libraries were screened to select highly potent siRNA leads, which were then validated through *in vitro* and *in vivo* experiments. Then, siRNA derivatives were designed to increase their stability and specificity via chemical modification (Figure 5). These derivatives were optimized through various analyses, and five siRNA candidates (HPV18 E6/E7 2 type, HPV16 E6/E7 3 type) were ultimately selected (not yet published). These candidates were tested for their *in vivo* therapeutic efficacy, characterized by siRNA pharmacokinetic/pharmacodynamic (PK/PD) analysis, and applied to siRNA pooling technology. These siRNA pools are likely to be highly effective in various combinations with conventional chemotherapies (CCRT) in alleviating tumors resistant to conventional treatments. In summary, future studies on HPV E6/E7-associated carcinogenesis and its cellular targets have significant implications for the development of potent therapeutic siRNA pools in combination with CCRT for cervical cancer, as well as HPV-associated cancers.

### HPV siRNA Nanoparticles for Targeted Anticancer Therapy

Over the last few decades, the applications of nanotechnology in medicine have been extensively explored in many areas, particularly in targeted drug delivery systems (DDS). The use of colloidal nanocarriers, including polymeric nanoparticles, polymeric micelles, and liposomes, is an emerging field of interest in nanomedicine. Compared with others, polymeric nanoparticles show great promise and are being widely investigated for application as synthetic vaccines, and delivery of siRNAs and oligonucleotides, antibiotics, and small molecule drugs [147]. The use of flexible polymeric chains in polymeric nanoparticles, such as PEG, are able to confer “stealth” properties to nanoparticles, because PEGylation of the nanoparticle surface prevents opsonin binding by reducing protein interaction. In addition, PEG-coated spherical nanoparticles with a neutral charge exhibit an increased half-life in the blood. Modulation of carrier properties promises more effective siRNA delivery to tumor sites, and could lead to enhanced drug safety and efficacy. Successful escape of siRNA nanoparticles from endosomes and the release of the contents into the cytoplasm are necessary to improve the efficiency of gene silencing [148]. Due to the acidic nature of endosomal/lysosomal vesicles, pH-buffering agents are widely exploited to promote cargo release. Under acidic conditions, various macromolecules with low-pKa amine groups have been shown to exhibit a “proton sponge effect.” When the complexes are internalized into the cell, these nanoparticle complexes are capable of buffering the endosomal vesicles, leading to swelling and lysis, thus releasing the nucleic acids into the cytoplasm. An alternate modification at the distal end of PEGylated nanoparticles with ligands against target cell surface receptors allows uptake via receptor-mediated endocytosis, leading to efficient intracellular siRNA nanoparticle release. To date, few siRNA delivery systems designed for anticancer treatment have reached the clinical trial stage, and the majorities are nanocarriers. The three leading RNAi candidates for cancer therapy were CALAA-01 (Calando Pharmaceuticals), ALN-VSP02 (Alnylam), and Atu027 (Silence Therapeutics) with enrollment starting in 2008–2009. These candidates are being used to clinically validate the three distinct systemic delivery platforms on which they were based: the cyclodextrin-containing polycation RONDEL technology, the AtuPLEX lipoplex technology, and SNALP liposomes. A small number of other DDS development companies, such as Bioneer (SAMi: Self-Assembled-Micelle-inhibitory-RNA), NanoCarrier Co., Ltd. (siRNA micelles), Celsion. EGEN, Inc. (siRNA-lipopolyamine nanoparticles), and Dicerna Pharmaceuticals, Inc. (EnCore) are involved in the process of investigating clinical DDS development. A detailed summary of clinical trials of siRNA-based therapeutics and their sponsor companies are listed in Table 3 [20,98,99,100,101,149,150]. Recently, Alnylam Pharmaceuticals in partnership with Tekmira developed a dual targeted RNAi drug in lipid nanoparticle carrier system, encapsulating two siRNAs to target mRNA of vascular endothelial growth factor (VEGF) and kinesin spindle protein (KSP) mRNA [151]. A better understanding of the intracellular fate of siRNA-nanoparticles will provide more rational guidelines for designing an ideal siRNA-nanoparticle delivery system for targeted cancer therapeutics. Currently, we are also in the process of developing candidate pooled siRNA nanoparticles, and conducting pre-clinical studies and clinical trials for the prevention and therapy of HPV-associated cervical carcinomas.

**Table 3:** RNAi-based therapies: Early stage clinical trials.

Candidate	Target	Focus of the study	Phase	Delivery	Sponsor
siRNA-EphA2-DOPC	EphA2	Advanced solid tumors	Phase 1 not yet recruited	Intravenous	M.D. Anderson Cancer Center
TD101	Mutant keratin	Pachyonychia congenita	Phase 1 completed	Intradermal	TransDerm
AGN 211745	VEGF receptor	CNV, AMD	Phase 2 terminated	Intravitreal	Allergan
Bevasiranib	VEGF	DME	Phase 2 completed	Intravitreal	Opko Health, Inc.
		AMD	Phase 3 withdrawn		
SV40 siRNA vectors	BCR-ABL	CML	Observational study completed	Hadassah Medical Organization
CALAA-01	M2 subunit of ribonucleotide reductase	Solid tumor	Phase 1 terminated	Intravenous, cyclodextrin	Calando Pharmaceuticals
siRNA IL-10	IL-10	Preeclampsia	Observational study terminated	National Taiwan University Hospital
SYL1001	Receptor TrpV1	Ocular pain dry eye	Phase 1 completed	Eye drop	Sylentis, S.A.
EZN-2968 Antisense oligonucleotide	HIF	Liver cancer or lymphoma	Phase 1 completed	Intravenous	Enzon Pharmaceuticals
ALN-VSP02	VEGF/ Kinesin spindle protein	Solid tumor	Phase 1 completed	Intravenous, SNALP liposome	Alnylam Pharmaceuticals
ALN-RSV01	RSV	Lung transplant patients/RSV infection	Phase 2 completed	Intranasal	
ALN-TTR02	Transthyretin	Amyloidosis	Phase 2 completed	Intravenous, SNALP liposome	
ALN-PCS02	PCSK9	Hypercholesterolemia	Phase 1 completed	Intravenous, SNALP liposome	
Miravirsen	miR-122	Hepatitis C virus	Phase 2 recruiting	Subcutaneous	SantarisPharma A/S
TKM-080301	Polo-like kinase-1	Advanced solid tumors	Phase 1 completed	Intravenous, SNALP liposome	Tekmira Pharmaceuticals
TKM-EBOLA	Viral RNA	Ebola infection (biodefense)	Phase 1 terminated		
PRO-040201	Apolipoprotein B	Hypercholesterolemia	Phase 1 terminated		
Atu027	Protein kinase N3	Advanced solid tumors	Phase 1 completed	Intravenous, AtuPLEXlipoplex	Silence Therapeutics
siG12D LODER	Mutated KRAS oncogene	Pancreatic cancer	Phase 2 / Phase 1 recruiting	Intratumoral	Silenseed, Ltd.
I5NP (QPI-1002)	TP53	ARF	Phase 1 completed	Intravenous naked siRNA	Quark Pharmaceuticals
		Kidney transplantation	Phase 1/2 completed		
PF-04523655	RTP801	DME	Phase 2 completed	Intravitreal	
QPI-1007	Caspase-2	Optic nerve atrophy NAION	Phase 1 completed	Intravitreal	Quark Pharmaceuticals
SYL040012	β-2 adrenergic receptor	Glaucoma, ocular hypertension	Phase 1/2 completed	Eye drop	Sylentis, S.A.
RXI-109	Connective tissue growth factor	Dermal scarring	Phase 2 Recruiting	Intradermal	RXi Pharmaceuticals
EZN-2968 Antisense Oligonucleotide	HIF-1	Advanced solid tumors with liver metastases	Phase 1 completed	Intravenous	National Cancer Institute
rHIV7-shI-TAR-CCR5RZ	Viral RNA and host factor	AIDS lymphoma	Phase 1 recruiting	Lentiviral	City of Hope Medical Center
Excellair	Syk kinase	Asthma	Phase 2	Inhalation	ZaBeCor
FANG vaccine	Furin	Advanced cancer, ovarian cancer, melanoma	Phase 1/2 recruiting	Plasmid	Gradalis, Inc.
CEQ508	β-catenin	Familial adenomatous polyposis (FAP)	Phase 1	Bacterial	Cequent Pharmaceuticals

VEGF: Vascular endothelial growth factor; DME: Diabetic macular edema; RSV: Respiratory syncytial virus; AMD: Age-related macular degeneration; CNV: Choroidal neovascularization; NAION: Non-arteritic anterior ischemic optic neuropathy; CML: Chronic myeloid leukemia; ARF: Acute renal failure; DME: Diabetic macular edema; PCSK9: Proprotein convertase subtilisin/kexintype 9; HIF: Hypoxia-inducible factor; SNALP: Stable nucleic acid lipid particle.

## 9. Conclusion and Future Perspectives

In spite of recent progress and various treatment modalities that have proved beneficial to some extent, no effective treatment is currently available for HPV-associated carcinogenesis. Even though the precise molecular targets have been characterized and several approaches for their inhibition have been demonstrated, not currently possessing an effective treatment approach constitutes a problem as important as finding novel methods for known targets and mechanisms. Nevertheless, the use of combinatorial treatment approaches with RNAi appears to be best suited for clinical protocols.

Since its discovery, RNAi has enabled interrogation of the role of individual genes in complex cellular processes. Advancements in RNAi-based screening technologies have fuelled anticipation of new discoveries. The siRNA pooling technique may prove advantageous, as there is an increased probability of at least one highly effective siRNA being present within the population. Moreover, the decreased concentration of each siRNA may reduce the potential for sequence-specific off-target effects. Another advantage over conventional therapies is that silencing of viral oncogenes and genes associated with carcinogenesis selectively enhance the chemosensitivity of tumor cells, which would help make attractive drug targets. However, siRNA-based therapeutics has encountered many barriers in clinical trials, including the stability of the RNA molecule itself, minimization of non-target effects, and limited choice of delivery systems for treatment. Although preliminary results from our study and several preclinical trials are just now becoming available, the results available to date support the safety of this innovative cancer therapy. All of these obstacles must be overcome if siRNA-based treatments for cancer are to be successful. In addition, associations and synergies between siRNA and other chemo/radio therapeutic agents may open new avenues for treatment of cervical cancer and improve the clinical outcome of patients with cervical cancer.
